# In Vitro Assessment of the Effects of Endodontic Sealers on Cell Viability, Adhesion, and Migration

**DOI:** 10.1111/aej.70013

**Published:** 2025-08-18

**Authors:** Anna Carolina Neves Leutz, Victor Augusto Benedicto dos Santos, Ana Cristina Padilha Janini, Amanda Nowicki de Salles, Brenda Paula Figueiredo de Almeida Gomes, Adriana de Jesus Soares, Talita Tartari, Marina Angélica Marciano

**Affiliations:** ^1^ Division of Endodontics, Department of Restorative Dentistry, Faculdade de Odontologia de Piracicaba (FOP) Universidade Estadual de Campinas (UNICAMP) Piracicaba São Paulo Brazil; ^2^ Division of Pharmacology, Anesthesiology, and Therapeutics, Department of Biosciences, Faculdade de Odontologia de Piracicaba (FOP) Universidade Estadual de Campinas (UNICAMP) Piracicaba São Paulo Brazil

**Keywords:** calcium silicate, dental cements, endodontics, epoxy resin‐based root canal sealer, fibroblasts

## Abstract

This study aimed to evaluate the in vitro cytocompatibility and bioactivity of the newly launched calcium silicate‐based sealer BioRoot Flow, compared with other calcium silicate‐based sealers (AH Plus Bioceramic Sealer and BioRoot RCS) and the epoxy resin‐based AH Plus Jet. NIH/3 T3 fibroblasts were used to assess cell viability, adhesion, and migration. Material characterisation was performed using SEM, EDS, and XRD. BioRoot RCS exhibited the highest cell viability, while BioRoot Flow showed fewer adherent cells and reduced migration at 72 h. EDS revealed zirconium content in BioRoot Flow, and XRD confirmed calcite presence in all calcium silicate‐based sealers. Variations in cell behaviour may be related to differences in chemical composition and surface properties, highlighting the need for further investigation.

## 1 Introduction

In endodontic procedures, the goal is the complete obturation of the root canal system to protect periapical tissues [1]. Calcium silicate‐based hydraulic sealers have become well‐established due to their antimicrobial properties, sealing ability, biocompatibility, and bioactivity [2, 3]. These materials, when used in vital pulp treatments, stimulate the proliferation of dental pulp stem cells [4, 5].

Cytocompatibility, biocompatibility, and bioactivity are key properties of calcium silicate‐based materials [6, 7]. Cytocompatibility refers to a material's ability to support cell viability and proliferation without inducing cytotoxic or cytostatic effects [8]. Biocompatibility extends this concept, encompassing proper cell adhesion and interaction with the surrounding environment. Cell adhesion plays a pivotal role in regulating differentiation, migration, and survival, making it a key indicator of how well a material integrates with biological tissues [9]. Bioactivity involves the material's capacity to actively promote biological responses, such as stimulating cell migration, which is critical for tissue repair and regeneration [10]. Thus, evaluating cell viability, adhesion, and migration provides essential insights into the biological performance of endodontic materials and their suitability for clinical use.

However, the development of new materials with different formulations can alter their biological properties, potentially compromising these attributes [11]. Assuming bioactivity and interaction with pulp or periapical cells, these materials are typically tested in vivo and in vitro to predict their clinical behaviour [12, 13, 14]. Cell adhesion is a good indicator of cytocompatibility, as this process plays a crucial role during periradicular repair and is closely related to cell viability, migration, and differentiation for tissue repair [15, 16, 17].

Since the biological properties of the newly launched calcium silicate‐based sealer, the ready‐to‐use BioRoot Flow (Septodont, Saint‐Maur‐des‐Fossés, France), have not yet been investigated, this study aimed to investigate its cytocompatibility and bioactivity in comparison to three established sealers: AH Plus Bioceramic, the powder/liquid BioRoot RCS, and the epoxy resin‐based AH Plus Jet. NIH/3 T3 fibroblasts were used as a biological model.

## 2 Materials and Methods

### 2.1 Endodontic Filling Materials

Four endodontic sealers were used (Table 1). The ready‐to‐use calcium silicate‐based sealers: BioRoot Flow (Septodont, Saint‐Maur‐des‐Fossés, France) and AH Plus Bioceramic (Dentsply, Konstanz, Germany); the powder/liquid BioRoot RCS (Septodont, Saint‐Maur‐des‐Fossés, France); and the epoxy resin‐based AH Plus Jet (Dentsply, Konstanz, Germany). The endodontic sealers were prepared according to the manufacturer's instructions under aseptic conditions within a laminar flow. Ready‐to‐use sealers already had an injectable paste; the powder/liquid sealer was mixed with a simple spatula no. 24 (Golgran, São Paulo, Brazil) on a smooth, polished 10 mm glass slab (Golgran, São Paulo, Brazil) until complete homogenisation of the powder and liquid components; and the paste/paste sealer already had a self‐mixing tip.

**TABLE 1:** Composition of endodontic sealers and batch number.

Material	Batch	Composition
AH Plus Bioceramic	KI230307	Zirconium dioxide, tricalcium silicate, dimethyl sulfoxide, lithium carbonate and thickening agents
AH Plus Jet	2204000437	Epoxide paste: bisphenol‐A epoxy resin, bisphenol‐F epoxy resin, calcium tungstate, zirconium oxide, aerosil and pigment Amine paste: 1‐adamantane amine N, N′‐dibenzyl‐5‐oxa‐nonandiamine‐1,9 TCDDiamine, calcium tungstate, zirconium oxide, aerosol and silicone oil
BioRoot Flow	B29973A	Tricalcium silicate, propylene glycol, povidone, calcium carbonate, aerosil (silica), zirconium oxide, acrylamide/sodium acryloyldimethyltaurate copolymer, isohexadecane and polysorbate
BioRoot RCS	Powder B29203 Liquid B29182	Powder: tricalcium silicate, zirconium dioxide and povidone Liquid: water, calcium chloride and polycarboxylate

### 2.2 Sample Preparation

The sealers were placed into teflon rings (Centerflon Ind Com Ltda, São Paulo, Brazil), measuring 5 mm in internal diameter and 2 mm in height, according to ISO 10993‐5 standards. After placing the sealers into the rings, they were incubated in an incubator at 37°C for 24 h for the initial setting of the materials. After 24 h, the teflon rings were removed from each mould and placed into 24‐well plates.

### 2.3 Cell Culture

Fibroblasts (NIH/3T3—CRL/1658) were obtained from ATCC (American Type Culture Collection) and cultured in DMEM (Dulbecco Modified Eagle Medium), supplemented with 10% Fetal Bovine Serum (FBS; Gibco, Grand Island, NY, USA) and 1% streptomycin/penicillin. Cells were maintained in an incubator at 37°C with 5% CO_2_; upon reaching approximately 80% confluence, cells were detached using 0.25% trypsin–EDTA solution (Gibco, Grand Island, NY, USA) for 5 min. The cells were plated at a concentration of 5 × 10^3^ cells per well in 96‐well flat‐bottom plates (TPP, Trasadingen, Switzerland). After plating, the cells underwent a 12 h adaptation period in the incubator at 37°C with 5% CO_2_.

#### 2.3.1 Cell Viability

The Thiazolyl Blue Tetrazolium Bromide (MTT; Sigma, St. Louis, MO, USA) assay was performed following ISO 10993‐5 standards. The assay included a negative control group containing only cell culture with DMEM and FBS, and all groups were tested in sextuplicate.

After the teflon rings were removed from each mould and placed into 24‐well plates, 1.5 mL of supplemented DMEM medium was added to each well to produce a cement sample eluate. These eluates were incubated at 37°C for 24 h. After the incubation period, the eluate from each group was removed for use in cell culture.

Cell viability was assessed 24 h after the start of treatment. After this period, the supernatant was removed, wells were washed with PBS, and 0.1 mL of MTT solution prepared at a concentration of 0.5 mg/mL was added to each well. The samples were then incubated for 4 h at 37°C. After the incubation period, the supernatant was removed, and the formazan crystals were dissolved in 0.1 mL of dimethyl sulfoxide (DMSO). Plates were agitated for 5 min and incubated for an additional 5 min for colour stabilisation.

Absorbance was measured using an automatic spectrophotometer (Asys UVM 340, Biochrom) at 540 nm to obtain absorbance values and subsequently calculate cell viability. Viability was expressed as a percentage, with the mean of the 6 wells from the experiments obtained in independent duplicates to ensure result reproducibility.

#### 2.3.2 Cell Adhesion

Five discs (2 mm in height and 5 mm in diameter) were fabricated from each cement. A suspension containing 5 × 10^4^ fibroblasts (NIH/3 T3—CRL/1658) was seeded directly onto the surface of each disc and incubated for 72 h. After incubation, the discs were fixed with 4% glutaraldehyde prepared in phosphate‐buffered saline (PBS) for 4 h. Dehydration was performed to the critical point using graded ethanol solutions (50%–100%, 20 min per step) and CO_2_ (Denton Vacuum DCP‐1), followed by drying and gold coating. Cell morphology was examined by scanning electron microscopy (SEM; JEOL JSM 5600 LV).

#### 2.3.3 Cell Migration

To evaluate the effect of different cement eluates on fibroblast migration, a horizontal migration assay was performed using NIH/3T3 cells. A total of 2 × 10^5^ cells/well were seeded in six‐well plates (*n* = 3). A linear scratch was created using a 200 μL pipette tip, and wells were rinsed with PBS to remove debris. Cells were then exposed to cement eluates at dilutions of 1:1, 1:2, and 1:4, or to culture medium alone (control). Wound closure was monitored at 24, 48, and 72 h. Images were captured at each time point, and the wound area was quantified using ImageJ software (NIH, Bethesda, MD, USA). Relative wound closure (RWC [%] = wound closure area [pixels] × 100/total area [pixels]) was calculated based on the area at 0 h. Migration was analysed in three intervals: 0–24 h, 24–48 h, and 48–72 h.

### 2.4 Structural and Chemical Characterisation

Structural and chemical characterisation of the calcium silicate‐based endodontic sealers was performed using scanning electron microscopy (SEM) and energy‐dispersive X‐ray spectroscopy (EDS). Samples were carbon‐coated and analysed under an SEM (JEOL JSM‐6610LV, Tokyo, Japan) at 1000× magnification in secondary electron mode. Elemental composition was identified and semi‐quantitatively determined by EDS.

### 2.5 X‐Ray Diffraction

Phase identification of the materials was performed using an X‐ray diffractometer (Rigaku, Tokyo, Japan), operating with Cu Kα radiation at 40 mA and 45 kV. The measurements were taken over a range of 2.5° to 70°, with a step size of 0.05° and a scanning speed of 1°/min. Phase identification was carried out using dedicated analysis software in conjunction with the ICDD database (International Centre for Diffraction Data, Newtown Square, PA, USA).

### 2.6 Statistical Analysis

Sample size calculation was performed using G*Power 3.1 software for Mac (Heinrich Heine University, Düsseldorf), based on data from previous studies [18]. For a total of three groups, an estimated standard deviation of 1.8 and a minimum detectable difference of 10 were used. A statistical power of 80% (*β* = 0.20) and a significance level of 5% (*α* = 0.05) were adopted. All assays were conducted in independent duplicates to ensure reproducibility.

Statistical analysis was performed using GraphPad Prism 8.0 software. Data normality and homogeneity of variances were assessed using the Shapiro–Wilk and Bartlett tests, respectively. For the cell viability analysis, a mixed‐model ANOVA (group × time) followed by Tukey's post hoc test was applied. For the cell migration data, two‐way repeated measures ANOVA with Tukey's post hoc test was used.

## 3 Results

### 3.1 Cell Viability

**FIGURE 1:**
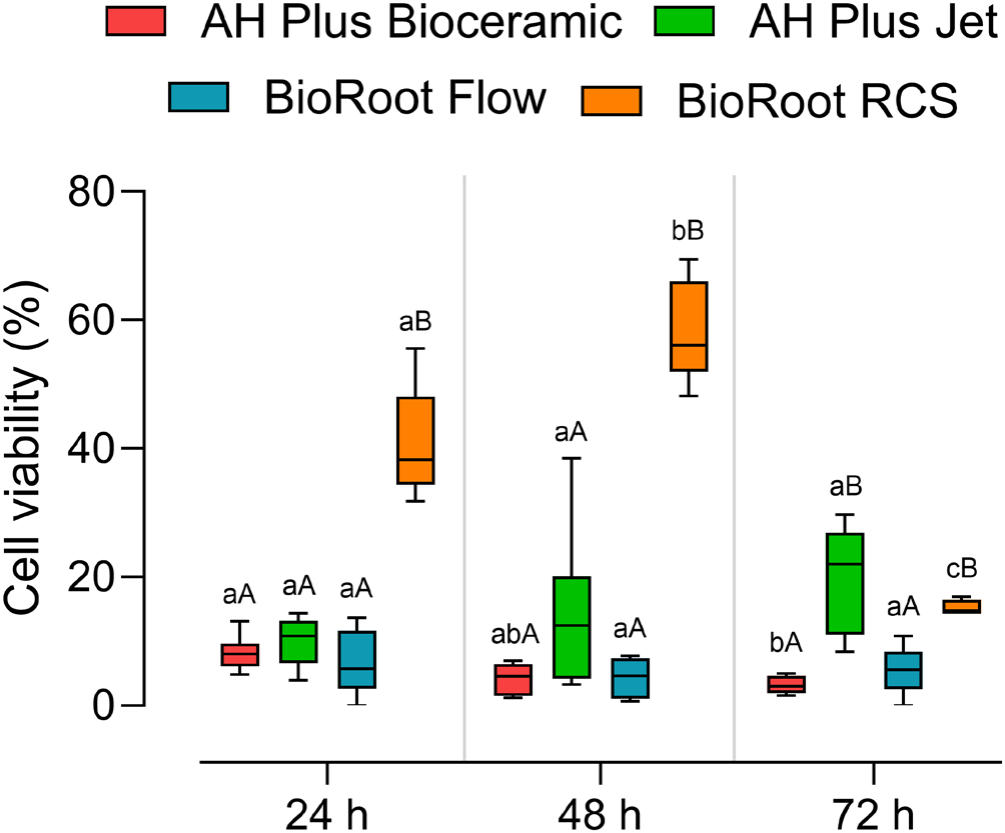
Multiple comparisons of the cell viability assay. Different letters indicate a statistically significant difference (*p* < 0.05). Lowercase letters represent comparisons between time points within the same cement, while uppercase letters indicate comparisons between cements at the same time point.

The analysis showed a significant difference for the time factor (*p* < 0.0001), cement factor (*p* < 0.0001), and the interaction between them (*p* < 0.0001). AH Plus Bioceramic showed a reduction in cell viability between 24 h and 72 h (*p* = 0.0416); AH Plus JET and BioRoot Flow showed no significant differences between time points (*p* > 0.05), whereas the BioRoot RCS group exhibited an increase from 24 h to 48 h (*p* = 0.0168), followed by a decrease from 48 h to 72 h (*p* = 0.0001). When comparing the sealers at individual time points, only BioRoot RCS showed a significant increase in cell viability compared to the other groups at 24 h and 48 h (*p* < 0.05). At 72 h, higher viability was observed in the BioRoot RCS and AH Plus JET groups (*p* < 0.05). The results are shown in Figure 1.

### 3.2 Cell Adhesion

**FIGURE 2:**
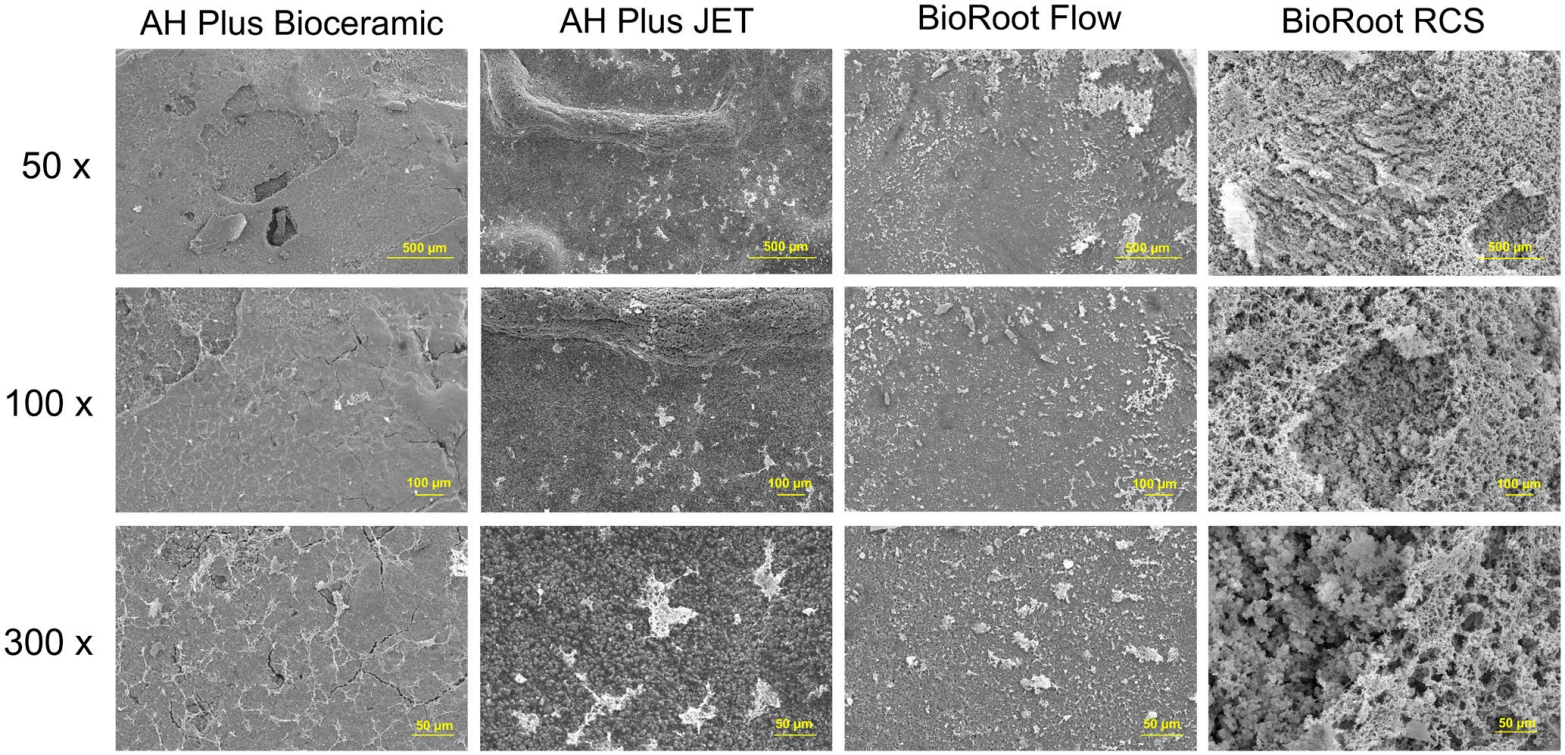
SEM analysis of NIH/3 T3 fibroblasts cultured on the surface of AH Plus Bioceramic, AH Plus Jet, BioRoot Flow, and BioRoot RCS discs after 72 h. Fibroblasts adhered to the surfaces of AHB and RCS discs exhibit a polyhedral morphology with extended dendritic projections. Scale bars are shown for 50×, 100×, and 300× magnifications.

As shown in Figure 2, the morphology and attachment of fibroblasts to the material surfaces were evaluated using scanning electron microscopy after 72 h. A moderate degree of cell attachment and spreading was observed on both AH Plus JET and BioRoot RCS. The cellular morphology indicated active adhesion, characterised by cytoplasmic extensions and a flattened profile. Fewer cells were attached to the surfaces of AH Plus Bioceramic and BioRoot Flow, which corroborated the findings of the MTT assay.

### 3.3 Cell Migration

**FIGURE 3:**
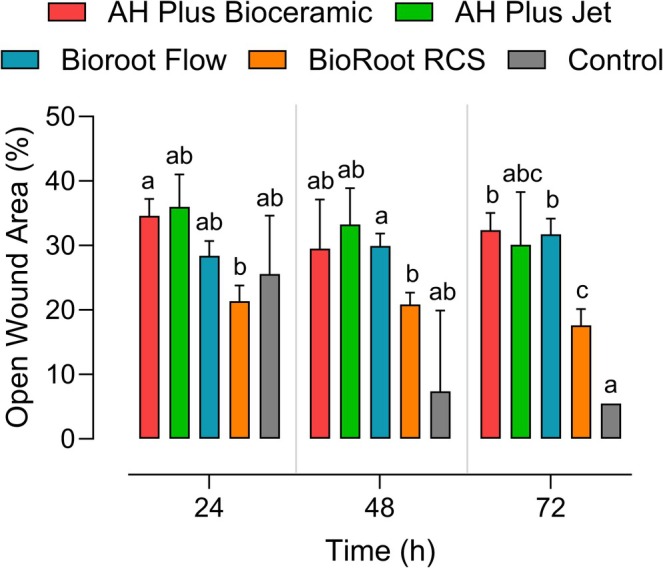
Multiple comparisons of the cell migration assay. Different lowercase letters indicate statistically significant differences (*p* < 0.05) between cements at the same time point.

**FIGURE 4:**
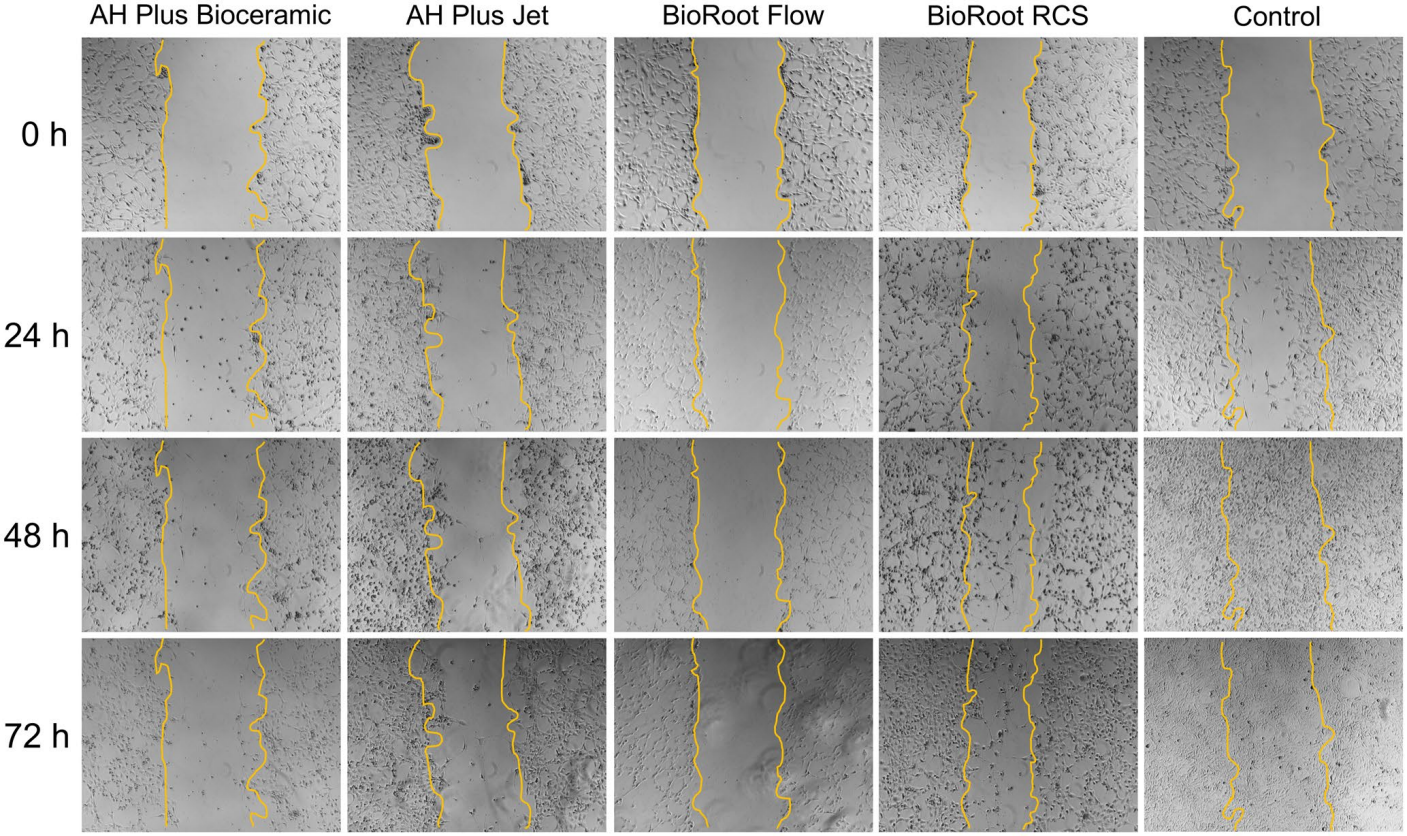
Scratch migration assay. Representative images showing wound closure in NIH/3 T3 fibroblasts following exposure to eluates from the four sealers at 24, 48, and 72 h.

The analysis revealed significant effects for time (*p* < 0.0141), cement type (*p* < 0.0004), and their interaction (*p* < 0.0162). At 24 h, only AH Plus Bioceramic and BioRoot RCS showed a significant difference between them (*p* = 0.0142), with no difference compared to the other groups. At 48 h, a significant difference was found between BioRoot Flow and BioRoot RCS (*p* = 0.0196), again with no difference relative to the remaining groups. At 72 h, BioRoot RCS showed significantly greater cell migration compared to AH Plus Bioceramic (*p* = 0.0108) and BioRoot Flow (*p* = 0.0111). These results are presented in Figures 3 and 4.

### 3.4 Structural and Chemical Characterisation

**FIGURE 5:**
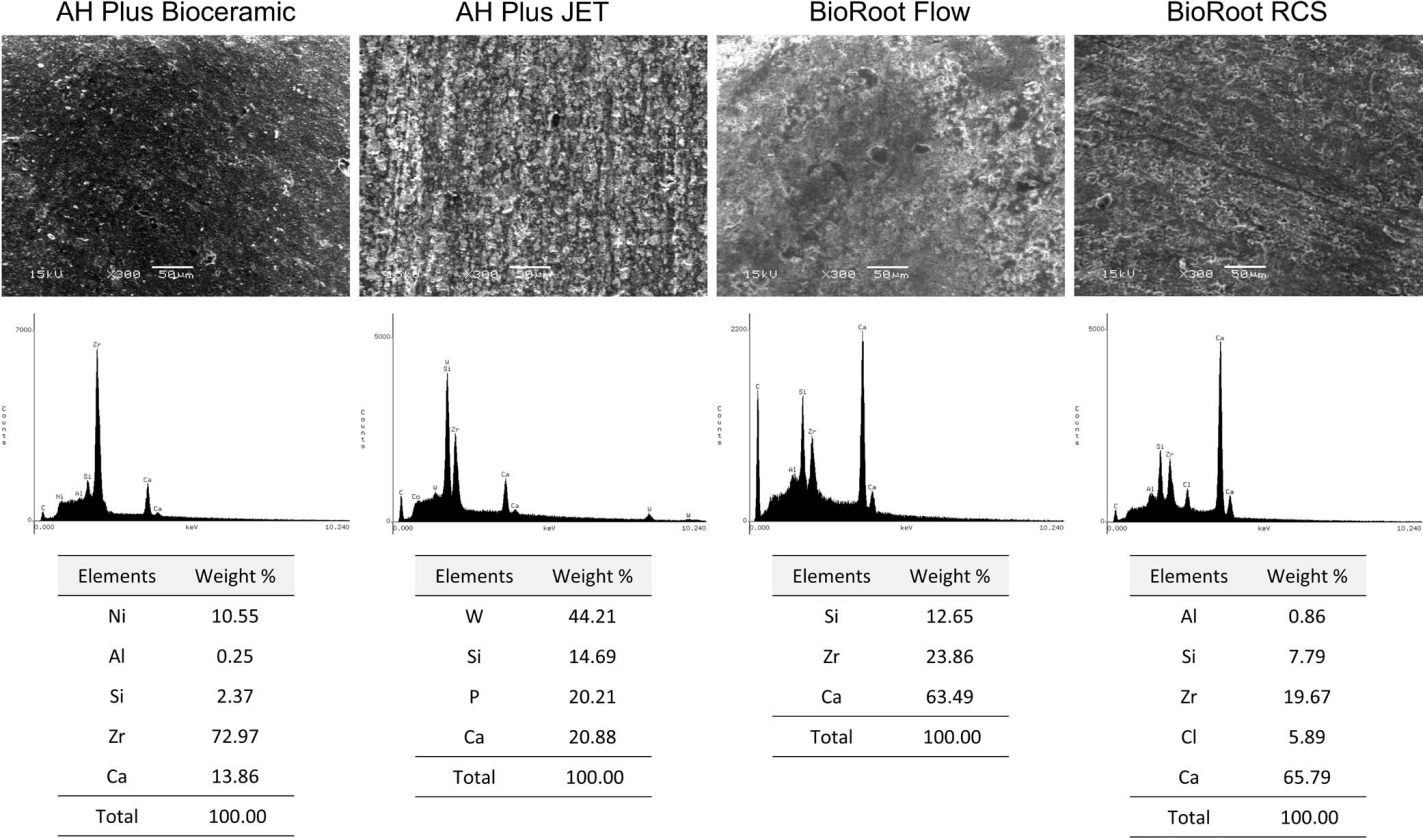
SEM‐EDS analysis. Elemental composition of AH Plus Bioceramic, AH Plus Jet, BioRoot Flow, and BioRoot RCS obtained via energy‐dispersive X‐ray spectroscopy.

SEM‐EDS analysis provided the qualitative and semi‐quantitative elemental composition of the surface of each material, as shown in Figure 5. The four sealers exhibited distinct elemental compositions. AH Plus Bioceramic showed a high concentration of zirconium (72.97%) and a relatively low calcium content (13.86%), along with the presence of nickel (10.55%). AH Plus Jet was characterised by high levels of tungsten (44.21%) and a balanced composition of calcium (20.88%), phosphorus (20.21%), and silicon (14.69%). BioRoot Flow demonstrated a high calcium content (63.49%) with moderate amounts of silicon (12.65%) and zirconium (23.86%). BioRoot RCS contained the highest calcium concentration (65.79%) and notable levels of chlorine (5.89%), along with lower amounts of aluminium, silicon, and zirconium.

### 3.5 X‐Ray Diffraction

**FIGURE 6:**
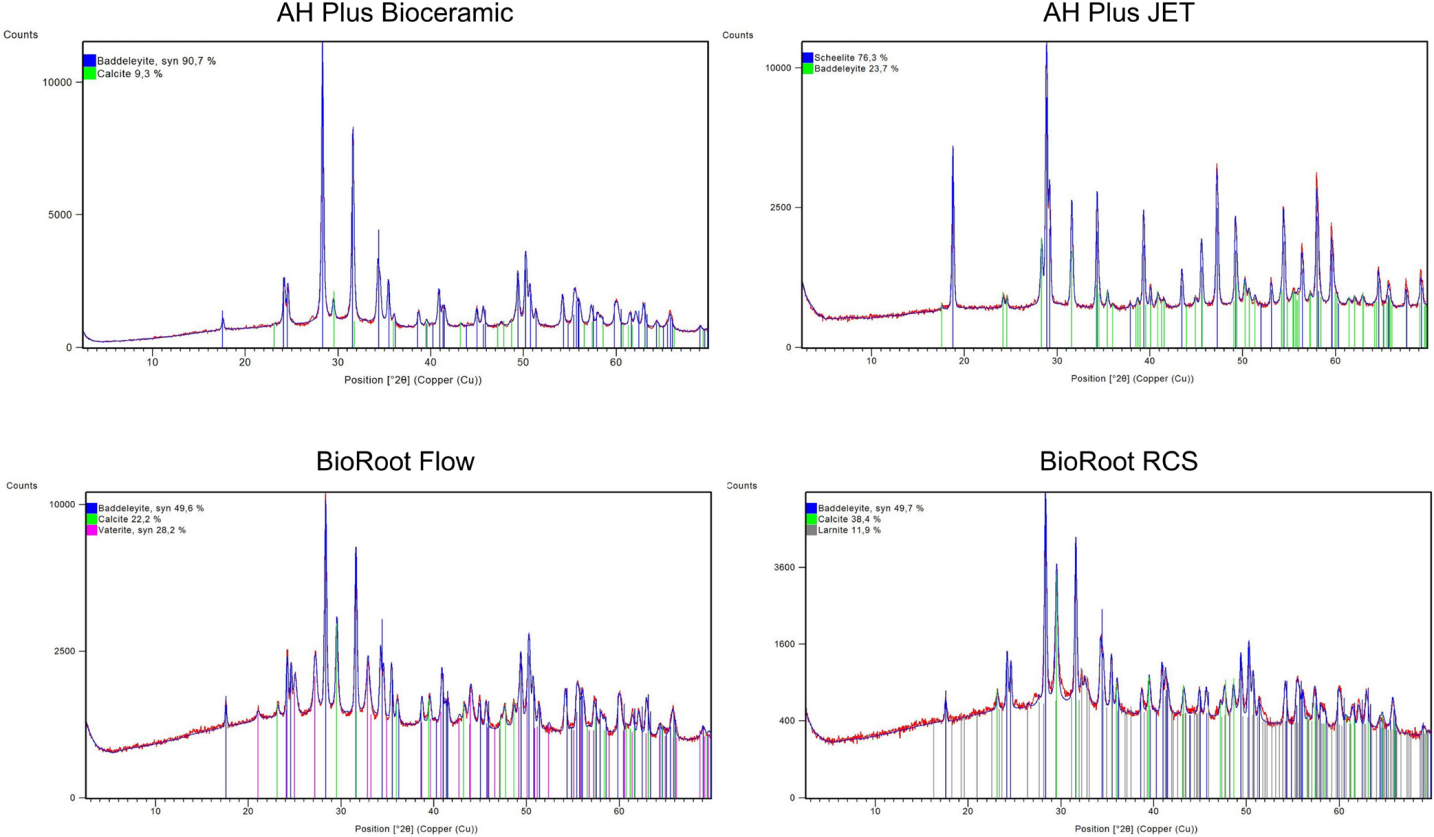
X‐ray diffraction patterns of the tested materials. All detected peaks were successfully identified using the COD (Crystallography Open Database).

The characterisation of the materials by X‐ray diffraction (XRD) is shown in Figure 6. The radiopacifiers Scheelite (CaWO_4_: COD 01‐077‐2233) and Baddeleyite (ZrO_2_: COD 01‐072‐1669) were detected in AH Plus Jet, as well as Baddeleyite in the materials AH Plus Bioceramic, BioRoot Flow, and BioRoot RCS. Additionally, calcite (CaCO_3_: COD 01‐072‐1651) was predominantly highlighted in the calcium silicate‐based endodontic sealers.

## 4 Discussion

This study evaluated the biocompatibility and physicochemical properties of four endodontic sealers: AH Plus Bioceramic, AH Plus Jet, BioRoot Flow, and BioRoot RCS, in terms of cell viability, adhesion, migration, and surface composition. The findings revealed significant differences among the materials, offering valuable insights into their potential clinical applications.

The BioRoot RCS group showed an initial increase in cell viability at 48 h, followed by a significant decrease at 72 h. This transient effect may be attributed to the absence of modulating agents such as propylene glycol present in BioRoot Flow [19, 20]. BioRoot RCS contains calcium chloride and polycarboxylate, which may lead to prolonged ionic release and sustained alkaline pH, resulting in delayed cytotoxic effects. The higher cell viability of AH Plus Jet across all time points may be explained by the epoxy resin‐based composition, which, despite its initial cytotoxicity due to amine byproducts, tends to stabilise quickly post‐polymerisation, reducing toxicity over time [21]. In contrast, AH Plus Bioceramic showed a progressive reduction in viability from 24 h to 72 h, possibly due to the presence of dimethyl sulfoxide (DMSO), which has been associated with both cytoprotective and cytotoxic effects depending on concentration and exposure time, and may contribute to delayed cytotoxicity [22]. This difference may be attributed to variations in formulation, including the presence of specific additives or differences in the release kinetics of bioactive ions, as suggested by the elemental composition analysis.

Scanning electron microscopy revealed clear differences in the adhesion behaviour of NIH/3 T3 fibroblasts on the surfaces of the sealers. Both AH Plus Jet and BioRoot RCS supported greater cell adhesion, as indicated by a flattened cell morphology with prominent cytoplasmic extensions, features characteristic of active interaction with the substrate. These findings align with the role of calcium ions in enhancing cellular attachment [16]. In contrast, AH Plus Bioceramic and BioRoot Flow exhibited fewer adherent cells, suggesting less favourable surface conditions for initial cell adhesion. The superior cell adhesion observed for AH Plus Jet may be related to its stable epoxy resin matrix, which limits the release of cytotoxic byproducts after polymerisation, promoting favourable surface conditions for cell attachment. In contrast, the higher pH and ionic release from tricalcium silicate in BioRoot Flow and AH Plus Bioceramic, along with additives like DMSO, may have interfered with early cell–material interactions, resulting in lower adhesion levels.

The chemical and structural analyses provided by EDS and XRD revealed distinct differences among the sealers, which may help explain the variations in their biological performance, as the highest calcium content was identified, which is essential for bioactivity but may also contribute to increased pH levels, potentially limiting cell viability and migration [23, 24, 25]. These differences in elemental composition and crystalline phases likely influence surface properties such as roughness, ion release, and pH modulation, which in turn affect fibroblast behaviour.

The release of calcium ions from calcium silicate‐based materials plays a key role in promoting cell–substrate interactions, favouring adhesion and migration processes [23]. The capacity of a material to support cell migration is crucial for tissue repair and is often enhanced by bioactive components. However, excessive calcium release can lead to a significant increase in extracellular pH, potentially compromising cell viability due to oxidative stress [24, 25]. These dual effects help explain the distinct cell migration patterns observed among the tested sealers. AH Plus Bioceramic did not promote increased migration and showed a progressive reduction in cell viability over time, possibly due to sustained calcium release and pH elevation. AH Plus Jet showed enhanced cell migration after 48 h, accompanied by increased viability, which may reflect a more balanced chemical environment due to its epoxy resin‐based composition. BioRoot Flow maintained stable migration and viability rates over time, indicating a moderate and possibly more cytocompatible ionic release profile. In contrast, BioRoot RCS induced an early increase in migration (from 24 h), likely due to a strong initial calcium ion release, but this effect declined by 72 h, paralleling the drop in viability, suggesting that the prolonged alkalinisation of the medium may have negatively affected cellular responses over time.

Altogether, the superior biocompatibility and cell migration‐promoting properties of BioRoot RCS support its use in clinical scenarios where tissue repair is essential. Its performance aligns with the growing clinical preference for bioactive sealers in contemporary endodontic practice. Although AH Plus Jet's favourable results in terms of cell adhesion and migration suggest it may be a suitable option in cases where a balance between mechanical stability and acceptable biocompatibility is desired; on the other hand, AH Plus Bioceramic and BioRoot Flow may require further optimization to enhance their biological performance.

Despite providing valuable comparative insights, this study has limitations. The in vitro conditions do not fully replicate the complexity of the in vivo periapical environment. Additional research, including animal models and clinical trials, is necessary to confirm these findings and evaluate the long‐term behaviour of these materials. Moreover, investigating the molecular mechanisms underlying their distinct biological responses may offer a deeper understanding of their clinical implications.

## 5 Conclusion

BioRoot Flow, possibly due to its lower calcium ion release and higher zirconium content, exhibited limited initial bioactivity. Nonetheless, it promoted cell migration, indicating potential for supporting cell recruitment in later stages of tissue repair. These findings underscore that, although calcium silicate‐based sealers possess bioactive properties, their early biological behaviour can differ significantly, which should be considered when selecting materials for clinical use.

## Author Contributions

**Anna Carolina Neves Leutz:** conceptualisation; methodology; investigation; writing – original draft; visualisation. **Victor Augusto Benedicto dos Santos:** conceptualisation; methodology; formal analysis; investigation; writing – original draft; visualisation. **Ana Cristina Padilha Janini:** conceptualisation; methodology; investigation; resources; writing – original draft; visualisation. **Amanda Nowicki de Salles:** conceptualisation; investigation; writing – review and editing. **Brenda Paula Figueiredo de Almeida Gomes:** conceptualisation; writing – review and editing. **Adriana de Jesus Soares:** conceptualisation; writing – review and editing. **Talita Tartari:** conceptualisation; writing – review and editing. **Marina Angélica Marciano:** conceptualisation; methodology; resources; writing – review and editing; supervision. All authors have contributed significantly; all authors are in agreement with the manuscript.

## Ethics Statement

The authors have nothing to report.

## Conflicts of Interest

The authors declare non‐financial conflicts of interest related to this study.

## Data Availability

The data that support the findings of this study are available from the corresponding author upon reasonable request.
